# Assembly, Annotation, and Comparative Whole Genome Sequence of *Fusarium verticillioides* Isolated from Stored Maize Grains

**DOI:** 10.3390/pathogens11070810

**Published:** 2022-07-20

**Authors:** Vishwambar D. Navale, Amol M. Sawant, Varun U. Gowda, Koteswara Rao Vamkudoth

**Affiliations:** 1 CSIR-National Chemical Laboratory, Biochemical Sciences Division, Pune 411008, India; vd.navale@ncl.res.in (V.D.N.); am.sawant@ncl.res.in (A.M.S.); 2 Academy of Scientific and Innovative Research (AcSIR), Ghaziabad 201002, India; 3 Theomics, International Private Limited, Bangalore 560038, India; varun@theomics.com

**Keywords:** *Fusarium verticillioides*, whole-genome sequencing, secretome, comparative genomics, plant-pathogen interaction, mycotoxin biosynthesis

## Abstract

*Fusarium verticillioides* is a plant pathogenic fungus affecting a wide range of crops worldwide due to its toxigenic properties. *F. verticillioides* BIONCL4 strain was isolated from stored maize grain samples in India, and produces high amount of fumonisin B1 (FB1). We report a comparative genomic analysis of *F. verticillioides*, covering the basic genome information, secretome, and proteins involved in host–pathogen interactions and mycotoxin biosynthesis. Whole-genome sequencing (WGS) was performed using the Illumina platform with an assembly size of 42.91 Mb, GC content of 48.24%, and 98.50% coverage with the reference genome (GCA000149555). It encodes 15,053 proteins, including 2058 secretory proteins, 676 classical secretory proteins, and 569 virulence and pathogenicity-related proteins. There were also 1447 genes linked to carbohydrate active enzymes (CaZymes) and 167 genes related to mycotoxin production. Furthermore, *F. verticillioides* genome comparison revealed information about the species’ evolutionary history. The overall study helps in disease prevention and management of mycotoxins to ensure food safety.

## 1. Introduction

*Fusarium* is a fungal genus that includes plant pathogenic species able to infect a variety of cereal crops, and causes diseases such as bakanae in rice; kernel, ear, and stalk rot in maize; and crown rot and head blight in wheat; all of which result in significant economic losses [1]. *Fusarium fujikuroi* species complex (FFSC) is a well-known group of plant pathogenic fungus that has the potential to produce mycotoxins in different agricultural products [2]. The FFSC contains phylogenetically distinct species, *F. verticillioides* (teleomorph, *Gibberella moniliformis*), *F. fujikuroi*, *F. proliferatum*, and *F. subglutinans*, that can infect agriculturally important crops such as maize, rice, sorghum, and beet [3], wheat and barley [4], cowpea [5], and a variety of ornamental crops [6].

*F. verticillioides*, *F. fujikuroi*, and *F. proliferatum* are the major contaminants of food grains in India and have the ability to produce different mycotoxins [7,8,9,10,11]. Divakara et al. [12] also reported sorghum samples contaminated with fumonisins (FUMs) producing *F. verticillioides* (33%) in Karnataka, Tamil Nadu, Maharashtra, and Rajasthan states, India. In addition, these species can cause various diseases to humans and livestock, such as keratitis, onychomycosis, sinusitis, invasive fusariosis and fusarial pneumonia [13,14]. Tupaki-Sreepurna et al. [15] also reported six species, including *F. verticillioides*, *F. sacchari*, *F. proliferatum*, *F. thapsinum*, *F. andiyazi*, and *F. pseudocircinatum*, from clinical isolates from southern India.

Focusing the attention on *F. verticillioides*, it is considered a major contaminant of a wide range of cereal crops and a producer of potent mycotoxins that adversely impact the supply chain [2]. *F. verticillioides* revealed a wide range of polymorphic nature and are randomly distributed throughout agricultural products [16]. Due to their heterogeneity, and pathogenic and toxigenic potential, the exact molecular mechanisms remain unknown. As a result, genome sequencing and comparative studies are crucial for the scientific community to understand pathway genes. Unfortunately, fungal research is a neglected area of research worldwide, especially in developing nations such as India [17]. Moreover, a range of patho-/sub-variants of fungi/microbes are evolving in the environment, causing various unanticipated diseases in different lifeforms [18]. In such a scenario, WGS is critical since it can aid in understanding the virulence of fungi.

Mycotoxins such as fumonisins (FUMs), fusaric acid (FA), beauvericin (BEA), and moniliformin (MON) are produced by FFSC members. These species can infect different food crops, produce mycotoxins, and cause chronic and acute toxicity to humans and animals [19,20]. However, FUMs toxin chemotypes produced by *F. verticillioides* are a major contaminant of maize globally, causing significant yield loss and posing a hazard to food safety [21,22]. Furthermore, FUMs have also been identified as a substantial contaminant of cereal crops, with disproportionately high concentrations in maize and rice [23]. Fumonisin B1 (FB1) leads to systemic toxicities such as hepatotoxicity, nephrotoxicity, neurotoxicity, and cytotoxicity [24]. FB1 and FB2 have been identified as potential carcinogens for humans and animals [25]. The consumption of FUMs-contaminated food increases the occurrence of esophageal and liver cancer [26]. Further, it has been found that FUMs also cause renal and hepatic toxicity and lead to tumor progression in rats. It acts as a ceramide synthase inhibitor, disrupts sphingolipid metabolism, and disturbs cell signaling and regulation [27,28].

Advancement in sequencing techniques has facilitated the development of reference genomes for identification, characterization, variation, and comparative studies [29]. The whole-genome sequencing (WGS) of fungi is also valuable for diagnosing diseases, preventing disease-causing fungi, and determining evolutionary relationships between fungal species. Illumina, Nanopore, and PacBio are some of the WGS technologies that yield high-quality sequencing data. Genome sequencing aids in advancing various fields, including medicine, agriculture, and other biotech sectors. *Fusarium* WGS also aids in the understanding of genome-wide variations, pathogenicity mechanisms, and genes involved in the secondary metabolite pathway [30]. The secretome of *F. verticillioides* and carbohydrate-active enzymes (CAZymes) assisted in identifying 166 proteins [31]. Only a few extensive studies are available on *F. verticillioides* in mycotoxin biosynthesis and pathogenesis [32,33]. However, there has been no detailed research on the complete secretome, repetitive elements (REs), and proteins involved in host–pathogen interactions in the *F. verticillioides* genome. To date, only six WGS of *F. verticillioides* are available at NCBI, and there are no reports from Indian subcontinents. To the best of our knowledge, only one report is available on the functional genome of *F. fujikuroi* from in India [10].

In our previous study, the *F. verticillioides* BIONCL4 strain isolated from a maize sample showed a more polymorphic nature, high FB1 production, and moderate-to-high pathogenicity (data not shown). However, there are no reports available on *F. verticillioides* WGS of Indian origin. Considering the above facts, the current study was conducted to understand the *F. verticillioides* genome and functions through genome-wide analysis. This study will help to understand the pathogenic and toxigenic behavior of the endanger *F. verticillioides*. In addition, it assists in developing disease resistance breeding programs by developing resistance genotypes against the pathogen.

## 2. Results

### 2.1. Genome Sequence, Assembly, Statistics, and Annotation

In our previous investigation, 60 strains of *F. verticillioides* were isolated from stored maize samples from 10 states in India, and their genetic diversity was studied using inter simple sequence repeats (ISSR) fingerprint. The selected *F. verticillioides* BIONCL4 strain produces a high amount of FB1, and showed moderate-to-high pathogenicity on root, shoot, and seed germination inhibition to maize genotypes (data not shown). Furthermore, to extend our previous study, WGS was performed to understand the molecular mechanisms of toxigenic and pathogenic potentials of the BIONCL4 strain. Based on the FB1 production and pathogenic potency of BIONCL4, WGS was performed using the Illumina NovoSeq platform; 1.3 Gigabases of HQ raw reads were obtained. Draft genome assembly using SOAPdenovo followed by assembly amelioration using Ragtag resulted in 42.91 Mb of the draft genome being submitted to the NCBI database under Bio-project PRJNA761025. BUSCO evaluation of draft genome completeness showed 98.50% coverage with the reference genome *F. verticillioides* 7600 (GCA000149555). Notably, the sequenced genome consists of 638 scaffolds, with a roughly N50 size of the scaffold being 4.23 Megbases (Table 1).

**Figure 1:**
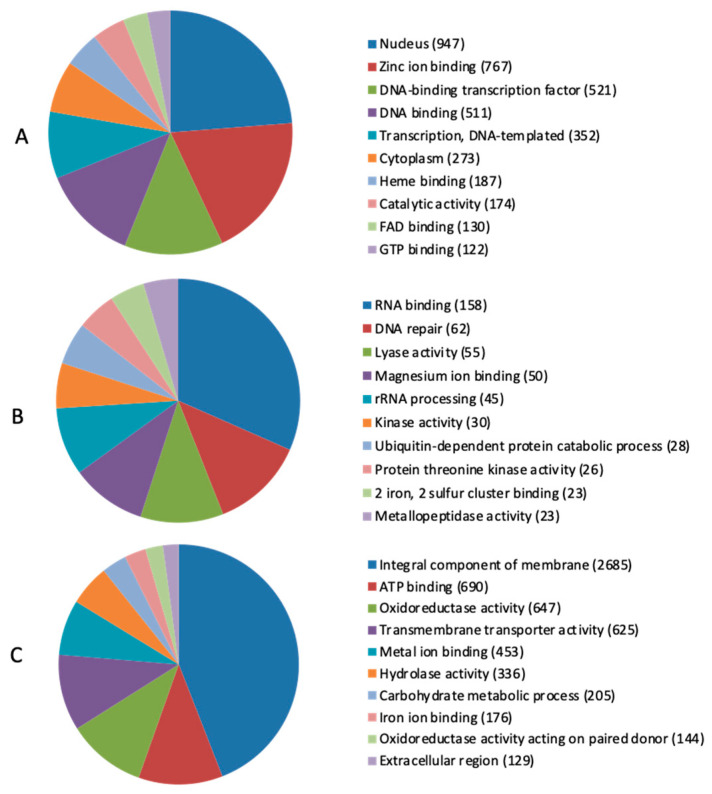
*Fusarium verticillioides* BIONCL4 functional annotation of genes based on gene ontology (GO) (**A**) biological processes; (**B**) cellular processes; and (**C**) molecular functions.

The protein-coding genes predicted from the assembled genome using the Glimmer and Genmark tools resulted in the identification of 15,053 protein-coding genes, conforming to the average gene density in the BIONCL4 genome of 37 genes per 100 kb to an average gene length of 3.72 kb. The total estimated GC content of the BIONCL4 genome was 48.24%. All the protein-coding genes CDS obtained by BLASTP were further processed for gene ontology prediction, resulting in 8845 genes annotated with gene ontology association (Figure 1, Supplementary Table S1).

### 2.2. Identification of Repetitive Elements (REs) and Simple Sequence Repeats (SSRs)

**Figure 2:**
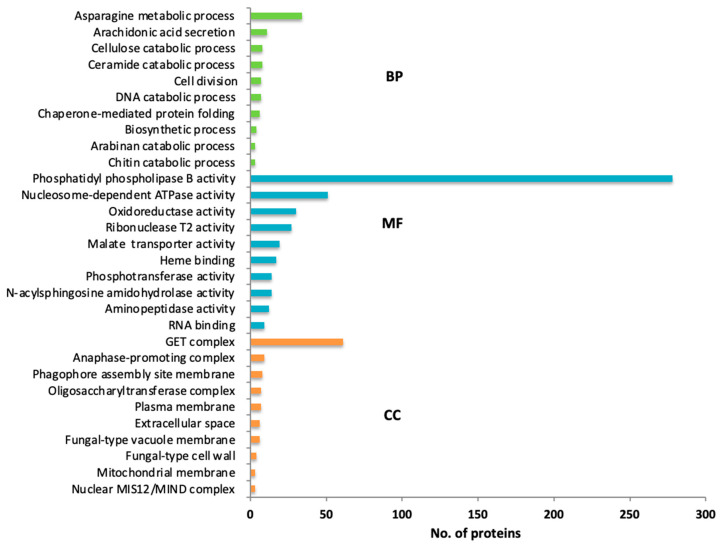
*Fusarium verticillioides* BIONCL4 functional annotation of secretome showing top hits of different proteins involved in molecular function (MF), cellular component (CC), and biological process (BP).

The BIONCL4 genome was analyzed for the presence of REs. A total of 378,085 bp of the sequence was identified, representing approximately 0.88% of the total genome. Different types of REs were also identified in the BIONCL4 genome, such as simple sequence repeats (SSR), which represent approximately 90.38%; small elements, 3.60%; retro-elements (SINE, LINE), 5.14%; DNA transposons (hAT-Charlie and TcMar-Tigger), 0.75%; and long terminal repeat (LTR) elements, 0.05%, respectively (Figure 2). Furthermore, 56 bp unclassified DNA elements, 20,823 bp total interspersed repeats, and 276 small RNA elements were observed (Table 2).

### 2.3. Secretome Prediction and Host–Pathogen Interaction Analysis

The secretion of fungal proteins mediates host–pathogen interactions by allowing the fungus to interact with its environment and host, and plays a critical role in its virulence. Out of 15,053 protein-coding genes, 2058 proteins could be identified as classical secretory proteins based on SignalIP v4.1 and targetP version 1.1 (Supplementary Table S2). Furthermore, gene ontology was used for the identified secretory proteins, which characterized them into three categories, biological process (91), molecular function (471), and cellular component (114) (Figure 2). The proteins involved in biological processes such as cellular metabolism and catabolism, cellular secretory pathways, chaperon-mediated protein folding, cell division, regulation of cellular processes, organelles compartmentalization, and cellular communications have highly corresponded to these categories. Proteins categorized in molecular functions allied with enzymatic activity, membrane transporter function, metal and iron-binding activity, complex macromolecules binding activity, and oxidation-reduction activity were mainly abundant. Proteins categorized in cellular components were part of a cell, organelle, and membrane, the structure of complex biomolecules, and their processing were more abundant.

**Figure 3:**
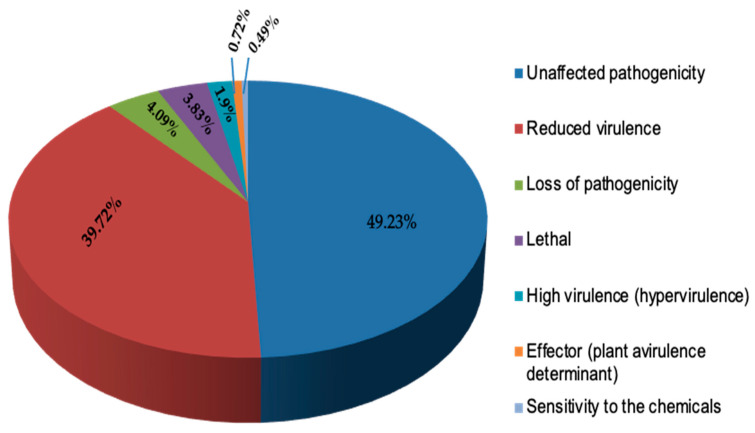
Functional annotation of *Fusarium verticillioides* BIONCL4 secretome genes showing top hits of different proteins involved in pathogen–host interactions (PHI-base) database.

Further, proteins involved in host–pathogen interaction were analyzed by the PHI database; among 2058 secretory proteins, 569 proteins have shown identity to the PHI-database belonging to different categories. Out of them, 39.72% proteins related to reduced virulence, 1.9% related to hyper-virulence, 4.09% proteins were loss pathogenicity, 3.83% were lethal proteins, 0.72% proteins related to plant virulence determinants, and 49.23% proteins were related to unaffected pathogenicity (Figure 3, Supplementary Table S3).

### 2.4. Identification of Carbohydrate-Active Enzymes (CAZymes) and Mycotoxin Biosynthetic Genes

**Figure 4:**
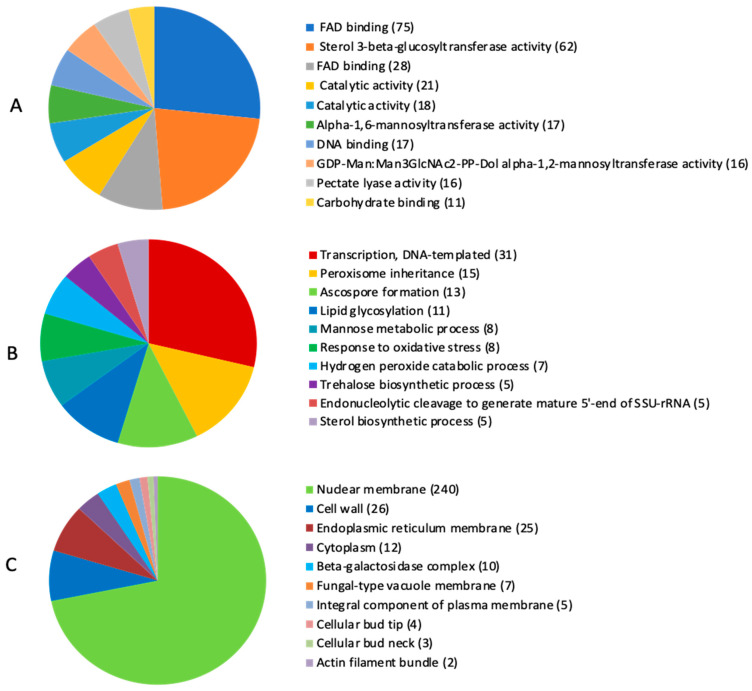
CAZymes identified in *Fusarium verticillioides* BIONCL4; (**A**) CAZymes in molecular function, (**B**) CAZymes in biological process, (**C**) CAZymes in cellular component.

It is well known that fungi cope with host-cell-wall polymers and access the saccharides that they use as a carbon source, which largely depends on the secretion of carbohydrate-active enzymes (CAZymes). Analysis of protein-coding genes using the CAZy database revealed 1447 genes related to the CAZy group. Apart from these enzymes, BIONCL4 secretome also consists of diverse types of oxidoreductases, transferases, hydrolases, and lyases. Based on the obtained data, it was found that the BIONCL4 secretome consists of a diversified nature of proteins which might play an important role in fungal colonization, nutrient acquisition, inactivation of host defense, and pathogenicity (Figure 4). Analysis of protein-coding genes and their potential role in mycotoxin biosynthesis resulted in identifying 167 genes associated with the biosynthesis pathway (Supplementary Tables S4 and S5).

### 2.5. Comparative Draft Genome Analysis of BIONCL4 Strain

**Figure 5:**
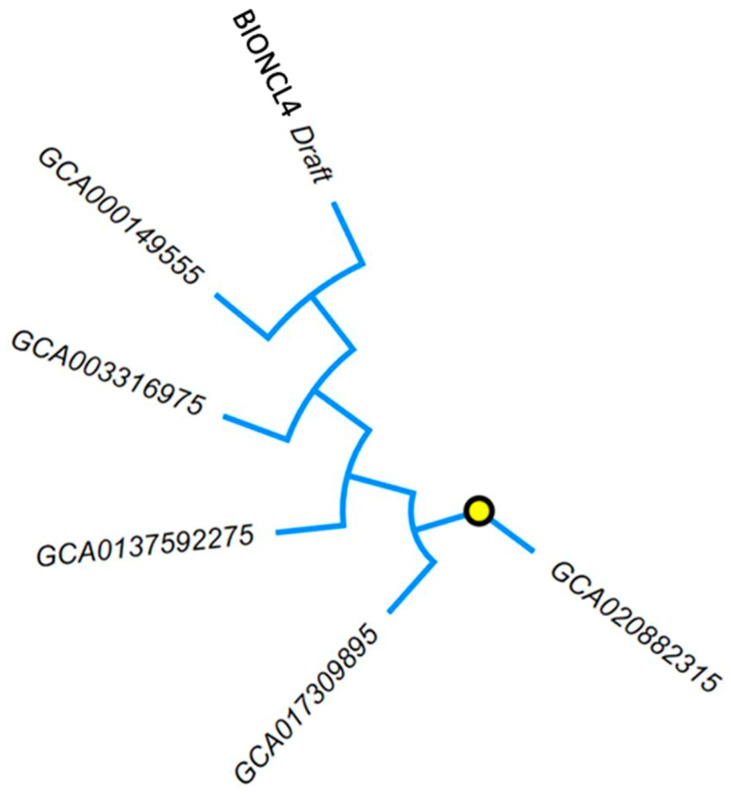
Whole-genome phylogenetic analysis of *Fusarium verticillioides* BIONCL4.

The phylogenetic tree, constructed by WGS of the selected isolates using ParSNP and visualized using the iTOLL web-based tool, showed clustering of BIONCL4 with *F. verticillioides* 7600 reference genome (GCA000149555) as compared to all the other isolates taken for genome comparison (Figure 5). However, the *F. verticillioides* strain isolated from maize (Italy) with genome assembly (GCA020882315) showed more distance with BIONCL4, when compared with different strains selected in this analysis. Interestingly, all the species’ WGS compared in this study were isolated from maize samples. Hence, our finding positively correlates the other studies reported on the genome of *F. verticilliodies.*

## 3. Discussion

India is primarily an agrarian nation, with a large rural population that depends on agriculture. However, various species of Fusarium infect agricultural commodities, and produce biothreat toxins that endanger both human and animal health. In the present investigations, *F. verticillioides* WGS was performed for understanding the functional genome for mycotoxins biosynthetic genes and virulence genes for the pathogenesis. To the best of our knowledge, only six *F. verticillioides* WGS are available at NCBI. However, there has been no detailed research on the *F. verticillioides* genome reported from an Indian origin [10,34]. This reveals a scientific chasm that may lead to significant plant and animal diseases. For example, *F. oxysporum f*. spp. *cubense* is the most severe tropical race 4 (TR4) that infects the Cavendish (AAA) group of bananas from the subtropical region of India [34]. Further, detailed phylogenetic relationships, virulence-associated effector genes, and race-specific molecular mechanisms of infection based on the presence of unique genes were studied [35]. Similarly, another draft genome was also published for the chickpea and chili anthracnose fungus, *Colletotrichum truncatum*, which helps to understand the molecular mechanisms and virulence genes and new genes responsible for disease development [36]. Unfortunately, no such efforts have been made in India to comprehend the disease severity and mycotoxin pathway study using WGS of *F. verticillioides*. This is the first report of *F. verticillioides* WGS from stored maize grains. In addition, the WGS obtained was compared to available WGS from the United States of America (USA), Australia, and others (Table 3). The WGS of the Indian isolate exhibits more sequence similarity to the strain from the American continent (GCA000149555). In the present study, *F. verticillioides* strain from Indian maize were processed for WGS. Further assembly and annotation were carried out using a hybrid approach, continued with Ragtag, BUSCO, and non-redundant (NR), Uniprot databases [33]. The obtained assembly size was 42.91 Mb, closer to the reference strain of *F. verticillioides* 7600 (41.84 Mb).

REs are mobile units that can propagate and expand in different regions of the genome; some of them are transposed with RNA intermediates known as retrotransposons, whereas some elements are directly transposed as DNA, called DNA transposons [37]. The REs present in the genome of *F. verticillioides* play an essential role in pathogenicity evolution due to variability in their sequence. Compared to other members of FFSC, *F. verticillioides* contains a low frequency of REs and agrees with reported *Fusarium* strains [38]. SSR plays a vital role in studying the polymorphism in *Fusarium* species [39]. In the BIONCL4 strain, 0.88% of REs represent the total genome. We analyzed SSR (90.38%), which is present and abundant in the BIONCL4 genome, followed by small elements, retro-elements, and DNA transposons, respectively, studied using RepeatMasker v4.0.9. The secretome plays an important role in understanding the mechanism of pathogenicity and interaction with the host. The abundance of proteins secreted by pathogens helps to understand the infection rate and severity. In the *F. fujikuroi* secretome, 1336 proteins were forecasted [40]. In *F. verticillioides* secretome, 151 proteins were reported, among which, 57 proteins are involved in cell wall degradation, and residual proteins take part in other cellular activities such as proteolysis, metabolism, defense, and response to various stresses generated at the cellular level [31]. In the current study, we predicted 2058 proteins in the BIONCL4 secretome; among them, 676 were predicted as classical secretory proteins based on SignalIP and TargetP. Furthermore, gene-ontology-based analysis showed the involvement of these proteins in cellular, molecular, and biological processes. The obtained results specified that the secretome of BIONCL4 strain comprised the diversification of proteins and their prearranged actions against plant defense to infect and cause disease effectively. *Fusarium* can produce different types of mycotoxins, ZEA, TRI, FUM, MON, and BEA [41,42]. *F. verticillioides* is well known for FUM production, in which 16 genes are involved, which code for proteins that lead to FUM biosynthesis and regulation, such as regulatory proteins, enzymes, and transporter proteins [43]. Researchers also reported that species of *Fusarium* are capable of producing FUMs in various food crops in India [10,12]. FUM biosynthetic genes with more than 97% identity were observed in the BIONCL4 genome, except three genes (FUM 10, 11, and 17), which suggests each species/strain may acquire an independent gene cluster [44]. Furthermore, ZEA is a polyketide mycotoxin produced by *Fusarium* species, and the genes ZEA1 and ZEA2 encode polyketide synthases involved in ZEA biosynthesis [45]. Surprisingly, ZEA1 and ZEA2 genes with more than 30% similarity were shown in the BIONCL4 genome, indicating that further validation may be required (Table 4). Thus, the CAZy database, PHI, and Kyoto Encyclopedia of Genes and Genomes (KEGG) gene ontology and homology findings may be used to analyze and explore the in-depth study linked to the pathogenicity and toxigenicity of *Fusarium* species (Table 4).

## 4. Materials and Methods

### 4.1. Culture Conditions and DNA Isolation

The *Fusarium verticillioides* BIONCL4 strain, isolated from contaminated maize grain samples collected from Andhra Pradesh, India, was processed for WGS. The culture was grown in a 250 mL Erlenmeyer flask containing 100 mL of sterile potato dextrose broth (PDB; pH 5.5) and incubated in a shaker at 150 rpm for seven days at 28 °C. After the incubation period, the vegetative mycelium was harvested by filtration. The obtained mycelium was ground into a fine powder using liquid nitrogen. About 100 mg of mycelium was used for the genomic DNA (gDNA) extraction using DNeasy Plant Mini Kit (Qiagen Hilden, Germany) as per the manufacturer’s instructions. The quality of gDNA was evaluated on a 0.8% agarose gel and was quantified on a Nanodrop spectrophotometer (Thermo Fisher Scientific, Waltham, MA, USA) and stored at −20 °C for future studies.

### 4.2. Genome Sequencing and Assembly

The quality of extracted gDNA of the *F. verticillioides* BIONCL4 strain was checked using agarose gel electrophoresis for integrity. Further, the gDNA was subjected to library preparation for deep sequencing as per Illumina’s recommended kit and protocol. Deep sequencing of the QC passed library was performed using the Illumina NovoSeq platform (Theomics International Pvt. Ltd., Bengaluru, India). Raw data quality control was performed using FastQC to check the read quality of the high-quality (HQ) filtered raw data. HQ reads with a Phred score above Q20 were chosen for further assembly. SOAPdenovo is a novel short-read assembly method that can build a de novo draft assembly for the eukaryotic genomes. The program is specially designed to assemble Illumina short reads. The assembly method includes the de novo assembly sequenced through a hybrid approach continued with Ragtag and BUSCO to assess the completeness of genome assembly. Assembly validation was performed using Bowtie-2 for aligning sequencing reads to long reference sequences and the Samtools package with default parameters. The quality and integrity of the genome were evaluated using benchmarking universal single-copy orthologs version 2 (BUSCO v2) with a fungal dataset on all the contigs.

### 4.3. Gene Prediction and Annotation

The total number of genes present in the genome of the BIONCL4 strain was predicted using Glimmer and Genmark. The predicted genes of BIONCL4 were functionally annotated with the help of the tBLASTx search tool against NCBI non-redundant (NR) and Uniprot databases with >30% identity and <1 × 10^−5^ cut-off E-values. Functional annotation of protein-coding genes for gene ontology and pathways was performed using BLAST2GO [46].

### 4.4. Identification of Repetitive Elements (REs) and Single Nucleotide Polymorphism (SNP)

The presence of repetitive sequences in the BIONCL4 genome, such as simple sequence repeats (SSRs), DNA transposons, retrotransposons including long terminal repeats (LTRs), etc., was characterized and identified using Repeat Masker v4.0.9 [47]. Sensitive mode runs with rmblastn version 2.2.27C RepBase update 20150807, RM database version 20150807 were used to identify repetitive families of repetitive sequences in the *F. verticillioides* genome. Mining of SSRs was performed using MISA software and categorized using standard parameters [48]. Furthermore, SNP in the BIONCL4 genome were studied using SNIPPY based on a BWA-mem/freebayes pipeline. SNPs (SAM tools) were annotated using SnpEff software by default parameters [49].

### 4.5. Secretome Prediction and Functional Genomic Analysis

The secretome analysis for BIONCL4 strain containing a set of 15,053 proteins was analyzed using SignalP v4.1 [50], TargetP version 1.1 [51], and Phobius [52] to forecast the secretory signal peptide in the secretome. Primarily, peptides containing more than 30 amino acids, SignalIP D-score = Y, 0.45 for 0 Tm/0.50 for 0.50 Tm as a cut off value, and TargetPLoc = S were merged. Further, the presence of transmembrane domain proteins was identified using TMHMM v2.0 [53]. The glycosylphosphatidylinositol (GPI)-anchored proteins forecasted by PredGPI [54] were processed for further investigations, and WoLFPSORT analysis was carried out with the help of “run WoLFPSORT v. 0.2” [55]. The PHI database was used to find out whether genes were involved directly or indirectly in pathogenicity and virulence [56]. The candidate pathogenicity and virulence-associated genes were identified by performing BLASTP searches of the *Fusarium* genome against PHI base version 4. The PHI base catalogs containing experimentally curated pathogenicity, virulence, and effector genes from different pathogens were used. Carbohydrate-active enzymes and protease families were screened through a local BLASTP search in the CAZy database [57] and Pfam with a threshold E-value and bitscore of 0.01 and 55, respectively.

### 4.6. Analysis of Orthologous Gene Families and Mycotoxin Biosynthetic Gene Identification

The comparative orthologous gene identification and analysis of four strains of *F. verticillioides* including BIONCL4 were studied using OrthoVenn of UC Davis with 1 × 10^−5^ and 1.5 as a default E-value and inflation value.

### 4.7. Comparative Phylogenetic Analysis of Fusarium Genomes

The Fusarium verticillioides BIONCL4 strain along with available *F. verticillioides* genomes with accession numbers GCA020882315, GCA017309895, GCA0137592275, GCA003316975, and GCA000149555 were subjected to comparative genome and phylogenetic analysis. The genome sequences of the comparing strains were retrieved from NCBI. The phylogenetic analysis of the WGS of *F. verticillioides* BIONCL4 with other phytopathogenic fungi was carried out using the ParSNP tool, and visualization using the iTOLL web resource.

## 5. Conclusions

The diverse and polymorphic nature of *F. verticillioides* is the major contamination of maize genotypes globally, and it produces various toxins chemotypes, including FBs, affecting the health of humans and animals. The comparative WGS showed that the BIONCL4 genome has the highest sequence similarity with the American continent *F. verticillioides* strain, followed by the Australian strain. Analysis of protein-coding genes revealed 1447 genes related to the CAZymes group and 167 genes associated with the mycotoxin biosynthesis. On the other hand, ZEA1 and ZEA2 are the key genes involved in ZEA biosynthesis, and about 30% sequence similarity was noticed. About 569 pathogenicity-related proteins were found out of 2058 secretory proteins. The comprehensive study provides valuable resources to design efficient resistant breeding programs against *F. verticillioides* pathogenicity in maize, and to manage FBs production in the food chain.

## Figures and Tables

**Table 1:** Whole genome sequence features of *Fusarium verticillioides* BIONCL4 strain.

Measurement	Details
HQ filtered raw data	8.6 Million reads
HQ filtered raw data (read count × read length)	1.3 Gb
Draft genome size (Mb)	42.91 Mb
Coverage	98.50%
Number of scaffolds	638
Largest contig	6.2 Mb
Average scaffold size	0.07 Mb
N50	4.23 Mb
Gaps	1198
(G + C) content	48.24%
Repeats	0.88%
Protein-coding genes	15,053
Average gene length (bp)	3.72 kb
Gene density	37 gene/100 kb
Secretory proteins	2058

HQ filtered raw data—high quality filtered raw data, Mb—megabases, Gb—gigabases, kb—kilobases.

**Table 2:** Identification of repetitive elements in *Fusarium verticillioides* BIONCL4 strain.

Repetitive Elements	Number of Elements	Length of Sequence (bp)	Percentage of Sequence (%)
Retroelements	391	27,767	0.06
Simple repeats	6873	290,113	0.68
DNA Transposons	49	3674	0.01
Small RNA	276	25,751	0.06
hAT-Charlie	6	442	0.001
TcMar-Tigger	5	399	0.001
Unclassified	1	56	-
LTR elements	3	213	-
ERVL-MaLRs	1	97	-
Total interspersed repeats	0	20,823	0.05
Low complexity	826	41,634	0.10

hAT-Charlie and TcMar-Tigger are DNA transposons, LTR—long terminal repeats, ERVL-MaLRs are long terminal repeats, bp—base pair.

**Table 3:** Comparative genome sequence features of *Fusarium verticillioides* strains from different geographic origins.

Features	*Fusarium verticillioides* Strains					
	BIONCL4	7600	BRIP14953	BRIP53590	NRRL20984	Fv10027_t1
Geographic origin	India	USA	Australia	Australia	USA	Italy
Isolation source	Maize	Maize	Maize	Maize	Maize	Maize
Assembly level	Scaffold	Chromosome	Chromosome	Chromosome	Scaffold	Contig
Gene bank assembly accession	------	GCA_000149555.1	GCA_003316975.2	GCA_003316995.2	GCA_013759275.1	GCA_020882315.1
Sequencing Method	Illumina HiSeq	shotgun sequencing	Illumina HiSeq	Illumina HiSeq	Illumina MiSeq	Illumina; Oxford Nanopore
Draft genome size (Mb)	42.91 Mb	41.84	42.54	42.29	41.92	44.65
Coverage	98.0x	-	90.0x	100.0x	50.0x	60.0x
Number of scaffolds	638	37	255	258	857	21
ScaffoldsN50(Mb)	4.23	1.95	4.02	4.02	0.10	2.91
GC content (%)	48.24%	48.68	48.15	48.26	48.80	47.90
Protein-coding genes	15,053	20,574	13,769	13,508	-	-

**Table 4:** FUMs biosynthesis genes involved in *Fusarium verticillioides* BIONCL4 strain.

Query ID	Identity (%)	Gene ID	Protein	Function (s)
1754_g	98.18	30058694	Acetyltransferase	FUM1_Highly reducing polyketide synthase, fumonisin biosynthesis
1746_g	98.38	30058700	Cytochrome P450 monooxygenase	FUM2_oxidoreductase activity, fumonisin biosynthesis
1752_g	99.71	30058695	Cytochrome P450	FUM6_Bifunctional_cytochrome_P450/NADPH--P450_reductase activity, fumonisin biosynthesis
1751_g	100	30058696	Dehydrogenase	FUM7_oxidoreductase activity, fumonisin biosynthesis
1750_g	98.93	5357319	Aminotransferase	FUM8_transferase activity, fumonisin biosynthesis
7269_g	34.92	30061262	Acyl-CoA_synthetase	FUM10_fumonisin biosynthesis
11438_g	55.05	30065034	Tricarboxylate transporter	FUM11_integral component of membrane, fumonisin biosynthesis
1745_g	99.46	30058701	NAD dependent epimerase/dehydratase	FUM13_oxidoreductase activity, fumonisin biosynthesis
1744_g	97.45	30058702	Non-ribosomal peptide synthetase	FUM14_ligase activity, fumonisin biosynthesis
1742_g	98.47	30058703	Acyl-CoA_synthetase	FUM16_ligase activity, fumonisin biosynthesis
10200_g	50.40	30064824	Sphingosine N-acyltransferase- like protein	FUM17_ integral component of membrane, fumonisin biosynthesis
6947_g	30.21	30061525	Non reducing polyketide synthase ZEA1	ZEA1_3-oxoacyl-[acyl-carrier-protein] synthase activity
13719_g	30.99	30066158	Highly reducing polyketide synthase ZEA2	ZEA2_3-oxoacyl-[acyl-carrier-protein] synthase activity, oxidoreductase activity zearalenone biosynthesis

## Data Availability

All data supporting the conclusions of this research manuscript are included in this manuscript and its additional files. The whole genome sequence project has been deposited at DDBJ/ENA/GenBank under Bioproject PRJNA761025.

## Supplementary Materials

The following supporting information can be downloaded at: https://www.mdpi.com/article/10.3390/pathogens11070810/s1, Table S1: The total number of genes of BIONCL4 and functional annotation of protein-coding genes for gene ontology and pathways; Table S2: The secretome analysis of BIONCL4 strain containing a set of 15,053 proteins was analyzed using SignalP v4.1, TargetP version 1.1 and Phobius; Table S3: Functional annotation of F. verticillioides BIONCL4 secretome involved in pathogen–host interactions (PHI-base) database; Table S4: Analysis of protein-coding genes using the CAZy database; Table S5: Analysis of protein-coding genes and their potential role in mycotoxin biosynthesis.
